# Feasibility and safety of left bundle branch area pacing in patients with surgical VSD closure

**DOI:** 10.1007/s10840-026-02332-8

**Published:** 2026-04-17

**Authors:** Enes Elvin Gul, Yerlan Turubayev, Omirbek Nuralinov, Ahmet Lutfu Sertdemir, Ahmet Taha Sahin, Muhammet Fatih Kaleli, Serhat Kesriklioglu, Sana Ouali, Mohammad A Ebrahim, Mohammad Salman Ghazni, Ugur Canpolat

**Affiliations:** 1 Department of Cardiology, Necmetin Erbakan University Hospital, Konya, Meram Turkey; 2 Interventional Arrhythmology Department, “University Medical Center” Corporate Fund, Astana, Kazakhstan; 3 La Rabta Hospital, Tunis, Tunisia; 4 Kuwait University, Kuwait City, Kuwait; 5 Madinah Cardiac Center, Madinah, Saudi Arabia; 6 Department of Cardiology, Hacettepe University, Ankara, Turkey

**Keywords:** Left Bundle Branch Area Pacing, Heart Block, Ventricular Septal Defect, Cardiac Pacing

## Abstract

**Background:**

Left bundle branch area pacing (LBBAP) provides more physiological pacing. Conduction continuity can be affected in patients with ventricular septal defect (VSD) closure due to patch material. We aimed to demonstrate the safety and feasibility of LBBAP in patients with surgical VSD closure.

**Methods:**

This is a multicenter, retrospective observational study including patients with surgical VSD closure who underwent LBBAP. Pre- and post-procedure data were included. Outcomes of the procedure have been demonstrated as well.

**Results:**

A total of 23 patients with surgical VSD closure were included. The median age was 17 years (range: 12–31). Baseline left ventricular ejection fraction was preserved in the majority of patients. Baseline QRS duration was 150 ms (122–178), which narrowed to a final paced QRS duration of 117 ms (111–126). LBBAP was successfully achieved in 19 patients (82%). Among successful cases, pacing types included non-selective LBBP in 43%, LVSP in 30%, selective LBBP in 9%. In cases where LBBAP could not be achieved, additional LV lead was implanted via the coronary sinus to improve ventricular synchrony. Procedure-related complications were infrequent and included one lead dislodgement and one late septal perforation.

**Conclusion:**

LBBAP can be safely performed in patients with surgical VSD closure with an acceptable success rate. In cases where LBBAP was unsuccessful, LOT-CRT may serve as an effective alternative to maintain ventricular synchrony.

## Introduction

Congenital heart disease (CHD) patients frequently require cardiac pacing due to conduction system abnormalities stemming from their underlying condition or surgical interventions [1]. Surgical repair of ventricular septal defects (VSD) often places the conduction system at risk of damage, either from direct injury or subsequent scarring, leading to pacing requirements. Traditional right ventricular (RV) pacing has historically been used in this population; however, it is associated with a potential risk of pacing-induced cardiomyopathy (PICM), mechanical dyssynchrony, and poor long-term outcomes [2].

Left bundle branch area pacing (LBBAP), a physiological pacing modality by directly engaging the cardiac conduction system, achieves superior electrical and mechanical synchrony compared to RV pacing and may mitigate the risk of PICM, especially in patients with preserved left ventricular ejection fraction (LVEF) [3]. Although left bundle branch area pacing has been shown to be feasible in selected congenital heart disease populations, existing evidence remains largely heterogeneous and lesion-nonspecific. Moore et al. have demonstrated that patients with congenital heart disease, including those with repaired VSDs, are particularly susceptible to PICM when exposed to chronic right ventricular pacing, underscoring the clinical importance of physiological pacing strategies [2]. Furthermore, while LBBAP has been reported as safe and effective in congenital heart disease, the presence of surgical patch material, septal fibrosis, and altered conduction system anatomy poses unique technical challenges, especially in patients with surgically repaired VSD [4]. In this subgroup, altered septal anatomy, fibrotic tissue surrounding the patch, and potential distal conduction system abnormalities may hinder stable conduction system capture. Consequently, data specifically evaluating the feasibility and safety of LBBAP in patients with surgically repaired VSD remain scarce, warranting dedicated investigation.

In this study, we aimed to evaluate the feasibility, safety, and short-term outcomes of LBBAP in patients with surgically repaired VSD while also exploring the role of left bundle branch–optimized cardiac resynchronization therapy (LOT-CRT) as an alternative pacing strategy.

## Materials and methods

### Study population

This multicenter retrospective study enrolled all available patients with surgically closed VSD who underwent LBBAP at the participating centers during the study period, based on institutional records. The study collected baseline patient demographics, echocardiographic parameters, concomitant congenital heart disease type, and pacing indications. In addition to LVEF, qualitative assessment of ventricular dimensions, septal thickness, and valve function were recorded, where available. QRS durations were measured from standard 12-lead ECGs using digital calipers. All measurements were independently overread by two experienced electrophysiologists blinded to the procedural outcomes. Baseline QRS duration was measured from intrinsic beats when available. In patients with complete heart block and no underlying escape rhythm, QRS duration was recorded during paced rhythm. Data collection was performed according to the guidelines of both local research ethics committees.

## LBBAP implantation

After obtaining verbal and written consent, patients were taken to the electrophysiology laboratory. LBBAP was performed using either stylet-driven lead (Solia S 60, Biotronik, Germany) or lumenless lead (SelectSecure 3830, Medtronic, Minnesota, US). The choice between a stylet-driven lead or lumenless lead was based on operator preference. In cases of initial failure with one approach, a crossover to the alternative system was attempted if feasible. Selectra 3 D delivery sheath (Biotronik, Germany) was used to deliver stylet-driven lead. However, the lumenless (Model 3830, 69 cm, Medtronic Inc.) pacing lead was introduced through a fixed curve (C315 HIS; Medtronic Inc.) sheath.

Once the sheath was advanced over the wire to the septum, a gentle counterclockwise torque on the sheath will “anchor” the sheath on the septum, allowing the guidewire to be removed from the sheath. The 12-lead ECG recordings and the intracardiac electrograms from the pacing lead were simultaneously displayed and continuously recorded on an electrophysiology recording system (Bard Medical system, Boston Scientific, US). Final 12-lead ECGs were obtained in all patients immediately post-implant to assess paced QRS morphology and duration. All patients underwent LBBAP, and the following procedural data were obtained: R-wave peak time in V5 or V6, V6-V1 interpeak interval, both unipolar and bipolar paced QRS durations, type of transition, and type of LBBAP (s-LBP, ns-LBP, LVSP, or DSP). Successful LBBAP was defined as evidence of conduction system capture according to established criteria (presence of r′ in V1, RWPT in V5/V6 < 85 ms, and V6–V1 interpeak interval > 33 ms) along with output-dependent QRS transition patterns demonstrating selective and non-selective left bundle branch capture. Selective LBB pacing (s-LBBP), non-selective LBB pacing (ns-LBBP), and LV septal pacing (LVSP) were considered successful LBBAP phenotypes. Deep septal pacing (DSP), which does not demonstrate conduction system capture, was classified separately and not considered successful LBBAP [3, 5].

If the LBBAP criteria were not met despite adequate lead depth in multiple locations in the septum or if the lead would not penetrate deep into the septum, then an additional left ventricular (LV) lead was implanted via the coronary sinus to improve left ventricular synchrony. Total fluoroscopy and procedure duration were also documented. A post-implant chest X-ray (PA and left lateral views) was obtained, and the patients were discharged the day after the procedure.

### Complications

Any LBBAP procedure-related complications were documented. Acute complications included pneumothorax, cardiac perforation, pericardial effusion, lead dislodgement, interventricular septal perforation, pocket-hematoma, device-related infection, transient ischemic attack and/or stroke, coronary artery injury, sensing issues, and an increase in capture threshold > 1 V. Also, chronic complications such as cardiac perforation, lead dislodgement, device infection, and significant changes in pacing parameters were recorded.

### Follow-up

The routine device follow-up was performed on the procedure day, at 1, 3, and 6 months, and 6-month intervals thereafter. During each follow-up visit, device interrogation was performed and pacing parameters were assessed. In addition, a 12-lead ECG was obtained and compared with the immediate post-implant ECG to verify paced QRS morphology and confirm the persistence of the pacing phenotype.

### Statistical analysis

A 1-sample Kolmogorov-Smirnov test tested the normality of the distribution of continuous variables. Continuous variables with normal distribution were presented as mean ± SD; non-normal variables were reported as median (min and max). Baseline and final paced QRS durations were compared using a paired samples t-test, with *p* < 0.05 considered statistically significant. Categorical variables were expressed as percentages and numbers. All analyses used IBM SPSS software version 25.0 (Chicago, Illinois).

## Results

### Study population

A total of 23 patients with surgical VSD closure were included. The primary pacing indication was complete atrioventricular block in 22 patients, most commonly occurring after congenital heart disease surgery. One patient with previously operated tetralogy of Fallot underwent device implantation due to atrial fibrillation associated with prolonged pauses. The clinical characteristics of the cases are depicted in Table 1. The median age was 17 years (range: 12–31). Fifteen patients were male. Surgical history included isolated VSD closure as well as concomitant congenital heart disease repairs, including ASD closure, PDA ligation, operated TOF, and complete AVSD (Table 1). Baseline QRS duration was 150 ms (122–178), which was narrowed with paced QRS duration of 117 ms. Baseline QRS duration was significantly reduced after pacing implantation (*p* < 0.001). Among the 23 patients, 12 underwent LBBAP as an upgrade procedure: eleven due to previously implanted epicardial or transvenous systems, and one due to RV lead fracture in a patient with an ICD. The remaining eleven patients underwent de novo LBBAP implantation. No LBBAP procedures were performed in the immediate postoperative period after VSD closure. Device follow-up was available up to 6 months for pacing parameters.

**Table 1:** Clinical characteristics of patients with surgical VSD closure

Number of patients	23	
Age, years	17 (12–31)	
Gender, male	15	
Rhythm, n (%)		
• CHB	22	
• AF	1	
Type of surgery		
• PDA closure	4	
• ASD closure	5	
• Operated TOF	3	
• Complete AVSD	5	
Baseline QRSd, ms	150 (122–178)	P value: <0.001
Final paced QRSd, ms	117 (111–126)	
Prior device, n (%)	13 (57)	

## Procedure Details

The details of the procedure are summarized in Table 2. Median procedure duration was 105 min (80–155), with a fluoroscopy time of 16.8 min (12–30). LBBAP was successfully achieved in 19 patients (82%). Procedural electrical parameters demonstrated a mean RWPT of 74.0 ± 15.6 ms and a V6–V1 interpeak interval of 38.1 ± 8.2 ms. Among patients with successful LBBAP (*n* = 19), pacing phenotypes included LVSP (30%), ns-LBBP (43%), and s-LBBP (9%). DSP was observed in four patients (18% of the total cohort) and did not meet the predefined criteria for LBB capture. While most procedures were performed as isolated LBBAP without additional LV lead implantation (82%), in patients where conduction system capture could not be achieved alternative pacing strategies such as LBBAP-CRT (9%) and LOT CRT (9%) were performed. Lumenless leads were used in 16 patients (70%), whereas stylet-driven leads were implanted in 7 patients (30%) (Table 2). Paced QRS morphology and duration were assessed at each follow-up visit and remained stable throughout the follow-up period in all patients.

**Table 2:** Procedure Details

Procedure duration, min	105 (80–155)
Fluoroscopy time, min	16.8 (12–30)
Lead, n (%)	
• LL	16 (% 70)
• SDL	7 (% 30)
RWPT V5/V6, ms	74.0 ± 15.6
V6-V1 interpeak, ms	38.1 ± 8.2
LBBAP success, n (%)	19 (82)
LBBAP type, n (%)	
• LVSP	7 (30)
• ns-LBP	10 (43)
• s-LBP	2 (9)
• DSP	4 (18)
Procedure type, n (%)	
• LBBAP	19 (82)
• LBBAP CRT	2 (9)
• LOT CRT	2 (9)
Complications, n	
• Lead dislodgement	1
• Late septal perforation	1

Electrical parameters showed no clinically relevant changes over time, with stable sensing, low and unchanged capture thresholds, and preserved lead impedance values throughout follow-up (Table 3). Procedure-related complications were rare and included one acute lead dislodgement and one late septal perforation; no life-threatening procedural complications were observed.

**Table 3:** Follow-up pacing parameters

	At implant	1-m FU	6-m FU
Sensing, mV	9.2 ± 4.7	10.7 ± 5.9	10.5 ± 5.6
Threshold @ 0.4 ms, V	0.7 ± 0.6	0.6 ± 0.4	0.6 ± 0.4
Impedance, Ohm	693 ± 153	647 ± 125	632 ± 123

**Fig. 1:**
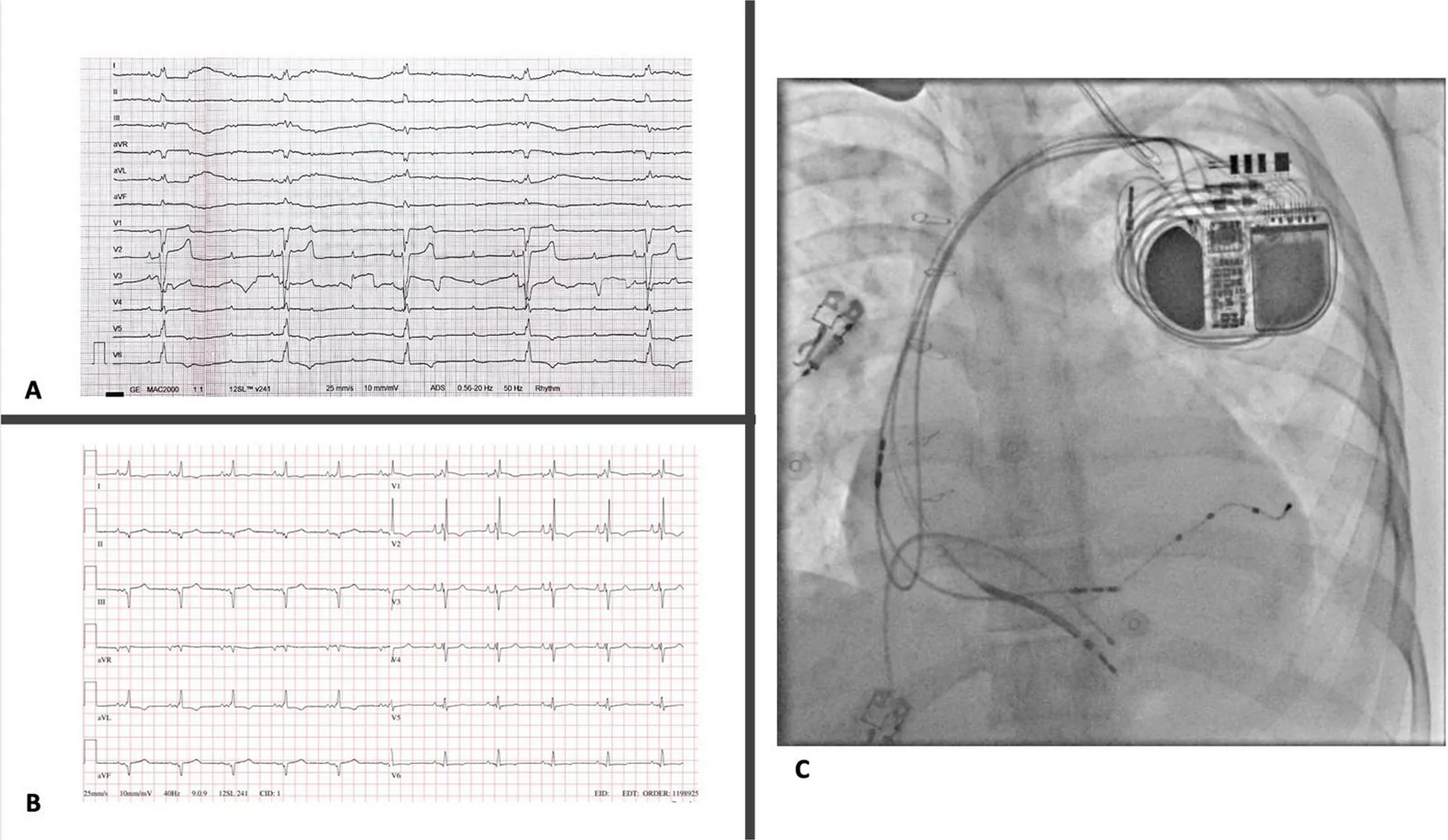
LOT CRT in a patient with failed LBBAP alone. A: Baseline 12-lead ECG showing 3:1 AV block and QRS duration of 130 ms. B: Final paced QRS showing RBBB pattern with left axis deviation and paced QRS duration of 122 ms. C: Final fluoroscopic view showing both LBBAP and LV lead in place

**Fig. 2:**
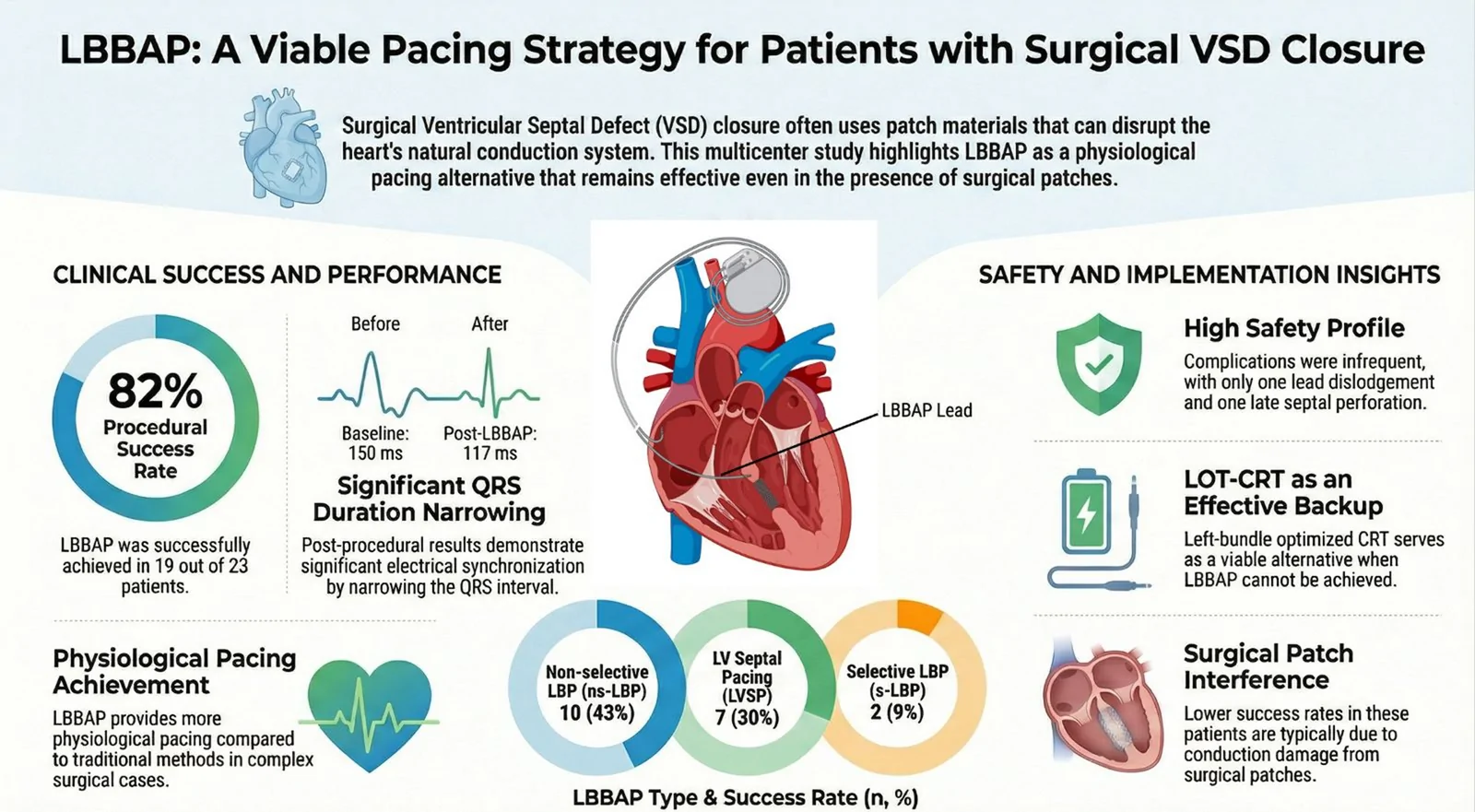
Illustration of VSD closure patch and LBBAP

Given the heterogeneous congenital heart disease background of the cohort, procedural success was descriptively evaluated according to the underlying diagnosis. Among the four patients in whom LBBAP could not be achieved, two had isolated VSD closure, one had a history of combined ASD and VSD closure with prior PDA ligation, and one had previously operated tetralogy of Fallot. Successful LBBAP was achieved in the remaining patients across different congenital substrates. Due to the limited number of patients within each subgroup, no formal statistical comparison was performed (Figs. 1 and 2).

## Discussion

In this multicenter cohort, we report the feasibility and short-term safety of LBBAP in patients with surgical VSD closure. The main findings of our research are as follows: (a) LBBAP was successfully achieved in more than 80% of patients with surgical VSD closure; (b) Electrical parameters were stable over the follow-up period; (c) Procedure-related complications were infrequent and limited to one acute lead dislodgement and one late septal perforation; (d) LOT-CRT can be offered as an alternative to LBBAP in case of failure to capture the conduction system.

Patients with CHD are at a higher risk of developing a pacing requirement compared with the rest of the population due to both underlying conditions and surgical interventions [4]. The conduction system runs postero inferior to the associated VSD, which, after surgical repair, can be inflamed or permanently damaged [6]. In the past, traditional transvenous or epicardial pacemakers have been implanted [7]. Traditional RV pacing is associated with the development of PICM, heart failure, and increased mortality in both CHD and non-CHD patients [4, 8, 9].

Left bundle branch area pacing is a promising physiological pacing modality that has been shown to promote better electrical and mechanical synchrony compared to traditional RV pacing and can prevent PICM in patients with preserved LVEF [10–12]. The role of conduction system pacing (CSP) in CHD was first assessed by O’Connor et al. [4] and found to be safe and feasible compared to patients with non-CHD. However, the main obstacle of LBBAP in patients with VSD closure can be faced during penetration of the ventricular lead. Capturing the conduction system can also be challenging or impossible in surgical scarring or patch around the conduction system. The presence of interventricular septal scar is one of the primary obstacles to achieving effective LBBAP and is linked to a poor clinical prognosis [13, 14]. It is important to acknowledge that achieving effective LBBAP in patients with surgically repaired VSD may be technically challenging due to factors such as septal patch material, dense fibrosis, and anatomical alterations of the conduction system. Furthermore, distal conduction disease may coexist and limit the ability to achieve reliable capture despite appropriate lead positioning.

The median fluoroscopy time in our study was 16.8 min. Although radiation exposure should be minimized, particularly in young patients with congenital heart disease, procedural complexity in this population may contribute to longer fluoroscopy durations. Factors such as septal patch material, altered septal anatomy, and the presence of fibrotic tissue may make septal lead penetration and optimal positioning more challenging. In addition, attempts to achieve conduction system capture or the need for alternative pacing strategies such as LOT-CRT may further prolong procedure and fluoroscopy times.

These factors highlight the need for individualized procedural planning and the potential utility of alternative pacing strategies in selected cases. In patients who do not respond to standard CSP due to septal scarring or the presence of patches disrupting the conduction system, His-optimized CRT (HOT-CRT) or LOT-CRT, administered as CSP alongside an LV epicardial lead, have been proposed as alternative methods to resynchronize the ventricles [15, 16]. LBBAP provides more reliable pacing electrical parameters for CHD patients than HBP [17]. Additionally, in patients with CHD, CSP was found to be non-inferior to conventional CRT concerning improvements in LVEF and a narrower-paced QRS width at one-year follow-up [17]. Although significant QRS narrowing was observed following LBBAP implantation, electrical resynchronization does not always translate directly into improved clinical or echocardiographic outcomes. In our cohort, systematic echocardiographic follow-up was not available across all participating centers, and therefore changes in left ventricular function or reverse remodeling could not be assessed.

Considering the relatively young age of our study population, the available follow-up period remains limited. Although pacing parameters and QRS morphology remained stable during the short-term observation period, longer-term follow-up is necessary to evaluate the durability of conduction system capture, lead performance, and potential long-term clinical benefits of LBBAP in this population.

### Limitations

Our study’s primary limitation was its retrospective and nonrandomized design. Prospective trials should be conducted to verify the long-term safety and clinical benefits of LBBAP in patients with VSD closure. The follow-up period was also limited; therefore, a more extended observation period is needed to evaluate the long-term benefits of LBBAP in this group of patients. The success rate of the procedure depended on the experience of the operator or center; therefore, differences among operators may have influenced the overall success rate. Another limitation was the absence of preprocedural imaging of VSD patch. Additionally, systematic echocardiographic follow-up was not available for all patients, and therefore changes in left ventricular function or ventricular remodeling could not be evaluated. More complex imaging modalities like cardiac MRI could be more helpful in this case. Anatomical heterogeneity among congenital heart disease patients and variable degrees of septal remodeling or fibrosis may affect the generalizability of our results. In addition, the follow-up duration was relatively short, which limits the ability to assess long-term pacing stability, lead performance, and clinical outcomes, particularly in this young congenital heart disease population.

## Conclusion

LBBAP appears to be a feasible and safe pacing strategy in patients with surgically repaired VSD based on short-term follow-up data. When conduction system capture cannot be achieved, combined with LV lead implantation may represent an alternative strategy to maintain ventricular synchrony. Nevertheless, larger prospective studies with longer-term follow-up are warranted to confirm the safety, efficacy, and durability of these physiological pacing approaches in this unique patient population.

## Data Availability

The datasets generated and/or analyzed during the current study are available from the corresponding author upon reasonable request.

## Declarations

### Use of AI Tools

No AI-assisted technologies (such as large language models, chatbots, or image generators) were used in the generation of the content or data analysis of this manuscript.
